# Prevalence and factors associated with adolescent pregnancies in Zambia: a systematic review from 2000–2022

**DOI:** 10.1186/s13690-023-01045-y

**Published:** 2023-02-20

**Authors:** Gift Malunga, Sidney Sangong, Farrukh Ishaque Saah, Luchuo Engelbert Bain

**Affiliations:** 1 United Nations Population Fund, UNFPA, UN House, Lusaka, Zambia; 2 ICAP Global Health, Columbia Mailman School of Public Health, Yaoundé, Cameroon; 3 School of Public Health, University of Health and Allied Sciences, Hohoe, Ghana; 4 Global South Health Research and Services, Amsterdam, Netherlands; 5 Lincoln International Institute for Rural Health, College of Social Science, University of Lincoln, Lincolnshire, UK

**Keywords:** Adolescent pregnancy, Teenage, Sexual and reproductive health, Socio-ecological model, Zambia

## Abstract

**Background:**

Adolescent pregnancy increases risk of short- and long-term adverse social and health outcomes for the adolescent mother and child. Zambia has high prevalence rates of adolescent pregnancy. However, the risk factors are varied and in need of further review and research. The study accordingly reviewed the prevalence and factors associated with adolescent pregnancy in Zambia.

**Methods:**

This systematic review was conducted following the 2020 Preferred Reporting Items for Systematic Reviews and Meta-Analysis (PRISMA) guidelines. The review included original peer-reviewed research articles published from 2000 onwards in English, retrieved from Medline, EMBASE, CINAHL, and African Journals Online databases. Thematic synthesis was used in the analysis of the data extracted from the included studies.

**Results:**

Six research studies carried out in Zambia (two quantitative, two qualitative, and two mixed methods) were reviewed and included. Prevalence of adolescent pregnancy in Zambia ranged from 29 to 48%. Additionally, it was found that 29.1% of the country’s adolescents, nationally, had given birth as of 2018. Factors at an individual’s level such as early or child marriage, exposure to media, knowledge about sexual and reproductive health (SRH) and contraception, contraceptive use, as well as risky sexual behaviours were found to be significantly associated with adolescent pregnancy. Peer pressure, educational attainment, household wealth, and the power dynamics of the household head were identified as the major socio-economic factors alongside socio-cultural, gender and sexual norms amongst other environmental and contextual factors. Policy level factors identified were lack and limited access to SRH information and services by adolescents, including an enabling legal environment.

**Conclusion:**

From the review, it was abundantly clear that a combination of individual, interpersonal, environmental, and an enabling legal/policy level factors significantly contribute to the high levels of adolescent pregnancy. There is a paucity of empirical research on the prevalence and determinants of adolescent pregnancy, which suggests an imperative need for large multi-site mixed methods studies to properly explore these and other determinants on a national scale, as well as the long-term implications of these pregnancies on adolescent mothers and babies. Multifaceted and multisectoral interventions which include improved access to education, economic empowerment, addressing gender and socio-cultural norms, should be implemented having due regard to the socio-cultural context which should ride on strong political will, failing which adolescent girls in Zambia will definitely be left behind.

## Plain English summary

Pregnancy increases risk of adverse short- and long-term outcomes for adolescent mothers and their babies. Adolescent pregnancy is significantly high in Zambia. However, the risk factors are varied and in need of further review and research. Using a systematic review process, we identify the trends and factors that influence adolescent pregnancy in Zambia from research articles published since 2000. We found that prevalence of adolescent pregnancy in Zambia was between 29%-48% and that nationally, 29.1% of the country’s adolescents had given birth as of 2018. Factors identified as influencing adolescent pregnancy in Zambia were analysed at four levels: 1) individual factors such as early or child marriage, exposure to media, knowledge about sexual and reproductive health and contraception, contraceptive use, as well as risky sexual behaviours; 2) interpersonal level factors such as peer pressure, educational attainment, household wealth, influence of male or female headed households, and family members; 3) community/environmental factors such as socio-cultural, gender and sexual norms; and 4) an enabling policy/legal environment covering mostly access to SRH information and services. It was emphasized that various stakeholders should deliberately collaborate to address these issues at all four levels using interventions that are socio-culturally suitable and should be supported by strong political will at policy and implementation levels to facilitate better access to education, improve economic empowerment of girls, review of gender and dysfunctional cultural norms and practices, amongst other considerations.

## Introduction

Adolescence is the transitional period from childhood to adulthood, accompanied by physical, psychological and emotional changes [1, 2]. Young girls often transition from childhood to adulthood with the onset of menarche, which marks the beginning of the initiation process in readiness for marriage [3, 4] among certain cultures in Zambia. During this period, many young people begin experimenting and engaging in sexual activities. Adolescent pregnancy, defined as pregnancy in girls aged between 10 and 19 years of age [5] is often an unfortunate offshoot of such sexual experimentation.

Adolescent pregnancy remains a key public health and development concern globally, especially in many low- and middle-income countries (LMICS). It is estimated that approximately 16 million girls aged 15–19 years, as well as an additional one (1) million below 15 years, give birth in LMICs [5]. Adolescent pregnancy has detrimental and far-reaching consequences for the majority of adolescent girls – for the rest of their lives –as it perpetuates poverty, deprives them of education, increases health risks and girl-child vulnerabilities while at the same time putting them in harm’s way and exposing them to violence [6]. In the result, adolescent pregnancy may have negative and unintended knock-on effects on many Sustainable Development Goals (SDGs), including; goal one (ensuring no poverty), goal two (zero hunger), goal three (good health and wellbeing including sexual and reproductive health), goal four (access to quality education), goal five (gender equality), and goal ten (reduction of inequality) [7].

The prevalence of adolescent pregnancy in Africa remains rather unacceptably high. For instance, a recent systematic review indicated that the prevalence of adolescent pregnancy was 18.8% in Africa as a whole, and 19.3% in sub-Saharan Africa [8]. Zambia, as indicated below, undoubtedly has one of the highest adolescent pregnancy prevalence rates in sub-Saharan Africa (SSA), conceivably with a significant attenuating effect on socio-economic development, riding on the fact that adolescents are a significant age group constituting 24% of the total population of Zambia [9]. Over the last five years, the rate of adolescent pregnancy has remained very high at 29.2%, with at least 35% of young girls in rural areas giving birth before, or by, the age of 18 years according to the 2018 Zambia Demographic and Health Survey (ZDHS) [10]. There are significant rural–urban variations in adolescent pregnancies in Zambia, with rural areas having an average of 37% compared to 17% in urban areas [10]. These averages potentially mask realities on the ground as some rural areas have adolescent pregnancy rates as high as 42.5% and 39.5% in Southern and Eastern Provinces respectively. Adolescent pregnancy is partly responsible for the high total fertility rate of 4.7 per woman and rapid population growth rate of 2.8% thus contributing about 20% of the total fertility rate [11].

Child marriages deprive adolescent girls of their sexual and reproductive health rights and curtails opportunities for them to realize their full potential and enjoyment of human rights entitlements as enshrined in various international treaties [6]. Child marriage prevalence in Zambia is one of the highest in the world, reducing marginally from 31.7% in 2014 to 29% in 2018 [12]. It is reported that 16.5% of girls aged 15–19 are married, while 31.4% of those aged 20–24 years got married before the age of 18 [13]. Major drivers of child marriage in Zambia include high poverty levels, limited access to quality education, limited life choices, and poor access to sexual and reproductive health services [14]. It is culturally accepted for marriages to take place between adolescents, to have intergenerational marriages, marriages of rectification of ‘shameful or dishonourable situations such as teenage pregnancy [6]. It has also been demonstrated that in some African patriarchal societies, power dynamics at play tend to reinforce child marriages [15, 16]. In order to protect girls’ rights and achieve progress towards national development, the Zambian government has prioritized ending child marriages through legal interventions and campaigns [6] anchored on the power of incumbency as the President of Zambia, H.E Hakainde Hichelema is the current African Union champion on ending child marriages.

The factors pertaining to poignant issues of adolescent pregnancy in Zambia are multifaceted, which suggests the need for multisector interventions to ameliorate the prevailing challenges, Unravelling and understanding these factors is key to the design and implementation of policies and interventions aimed at curbing adolescent pregnancy. This review aimed to assess the prevalence of adolescent pregnancy with recourse to Bronfenbrenner’s Socio-ecological Model (SEM) as the main conduit towards systematically identifying critical factors impacting adolescent pregnancy. The SEM is a pivotal theoretical framework which anchors robust examinations, investigations and research endeavours prescribed by the multiple factors at different sociodemographic levels that are associated with adolescent sexual and reproductive health (ASRH).

## Methods

### Study design and search strategy

This systematic review was conducted following the 2020 Preferred Reporting Items for Systematic Reviews and Meta-Analysis (PRISMA) guidelines [17]. We combined text words and medical subject headings such as pregnancy OR birth OR delivery with adolescent OR teen to search Medline, EMBASE, CINAHL, and African Journals Online from the database inception to 10 October 2021. The reference lists of eligible full texts were hand searched to retrieve articles missed during the search.

### Study selection

Primary studies (both qualitative and quantitative) that reported, or contained relevant data, on the prevalence and factors associated with adolescent pregnancy in Zambia were considered for inclusion in this review. The study excluded reviews, editorials, case reports, and case series with fewer than 30 participants. Detailed inclusion and exclusion criteria are presented in Table 1. We exported citations retrieved from database searches to EndNote X9 for removal of duplicates, and the (re)-duplicated records were uploaded to Rayyan QCRI for screening based on title and abstract.

**Table 1:** Inclusion and exclusion criteria

Inclusion criteria	Exclusion criteria
Peer-reviewed publications	Reviews and editorials
Published in English language	Case reports and case series with less than 30 participants
Primary studies published since the year 2000	
Population is adolescents (10–19 years)	

### Outcome measure

Adolescent pregnancy was defined as pregnancy or childbirth occurring in women less than 20 years old. This included self-report or evidence (record) of childbirth at a health facility by women aged less than 20 years. The prevalence of adolescent pregnancy was calculated as the number of women who become pregnant or had a child while they were less than 20 years old.

The second outcome of this study focused on factors influencing adolescent pregnancy. Factors associated with adolescent pregnancy, in the quantitative study conducted, were determined from the p-value less than 0.05 for the statistic from the final inferential analysis carried out. Themes related to factors with a bearing to adolescent pregnancy identified from the findings of the included qualitative studies were collated.

### Quality assessment and risk of bias

Quality assessment of the six (6) papers was carried out using Sirriyeh et al.’s tool for quality assessment of studies with diverse designs (QATSDD) [18]. The use of this tool also allows for comparisons of the qualities of quantitative, qualitative, or mixed-methods papers within the same domain field of study. Two authors (FIS and LEB) independently assessed the included studies and where disagreements were encountered, a third author resolved this through a third assessment informed by discussions held. The tool awards quality score on a four-point scale from 0 to 3 with accompanying guidance notes for using the scoring criteria (qualitative study (14), quantitative study (14), and mixed methods (16)) [18]. The criteria included a theoretical framework, a statement of aims and objectives, a description of the research setting, sample size in relation to analysis, representativeness, sample size, data collection procedures, rationale for choice of data collection tool, recruitment data, statistical assessment of reliability and validity of tool (quantitative only), fitness between stated research question and method of data collection (quantitative only), stated research question and format and content of data collection tool (qualitative only), stated research question and method of analysis, justification for selected analytical method, reliability of analytical process assessment (qualitative only), and user involvement in design, and discussion of strengths and limitations [18].

Each research paper was awarded a score per criterion and index scores generated. The total quality scores of the included studies were computed in percentage using the expected total scores (42 for quantitative and qualitative studies and 48 for mixed methods studies). Percentage scores greater than 50% were designated as high while those less than or equal to 50% were graded low-quality.

### Data extraction and analysis

Two authors independently extracted data on the surname of the first author, year of publication, and determinants of adolescent pregnancy. The data extraction tool contained information on the author and year of publication, study aim, study design, study area and province, sample size, study population, and factors associated with adolescent pregnancy. A thematic synthesis was adopted in this review. The analysis of the factors influencing adolescent pregnancy was based on themes from the SEM. As such the themes for determinants of influence on adolescent pregnancy were categorized into individual (for example, exposure to media, and knowledge on use of contraceptives), interpersonal/socio-economic (for example, educational status, household wealth), and policy /legal framework (for example, knowledge of and access to sexual reproductive health services) factors. Bronfenbrenner’s model is used because it allows for an assessment of all possible factors that contribute to a phenomenon, addressing the issue as resulting from influences of multifaceted and multilevel factors [19].

## Results

### Search results

**Fig. 1:**
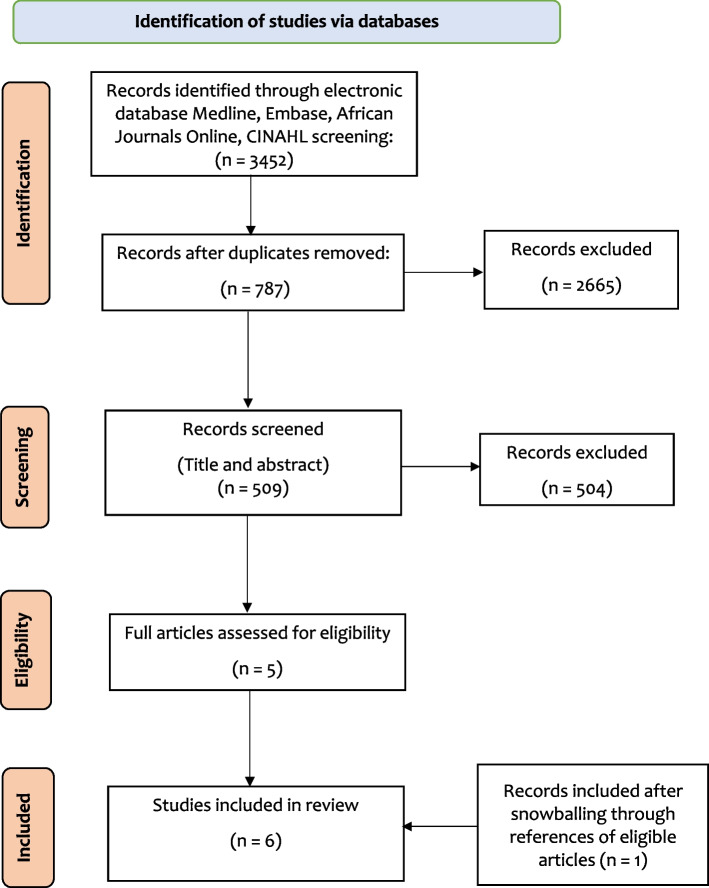
PRISMA flow of the search strategy

A total of 3,452 papers were initially accessed from various electronic database searches and digital library catalogues, however 2,665 research had to be eliminated since they were duplicates. The remaining articles’ titles and abstracts were then evaluated, and 504 studies were reviewed and removed because they were not germane to the study. On the basis of the established inclusion and exclusion criteria, the eligibility of the remaining five (5) full-text publications was assessed. From snowballing through references of eligible articles, one (1) study was further assessed and included. Thus, in the final analysis, only the six papers that met the eligibility requirements were included (Fig. 1).

### Overview of included studies

Of the 6 studies retained in the final review, two (2) were qualitative, two (2) were mixed methods and two (2) were cross-sectional. The studies were carried out in the following Provinces of Zambia: Southern (2), Central (2), Eastern (1), North-Western, as well as in Lusaka (2) and Copperbelt (1) while two used national-level data from the Zambia Demographic and Health Surveys (ZDHS). All the six studies in the provinces showed high quality ranging from lowest 54.7% [20] and 76.2% [21] (Table 2). Guided by Wado et al.’s [21] study, all the five did not have explicit theoretical frameworks. Additionally, all the six studies did not provide any justification for the choice of data collection tools and all the quantitative and mixed methods studies did not assess statistical reliability and validity of the tools, which could be due to the use of secondary data for some of the studies [21, 22]. Such studies did not select the data collection tools and conducted their own data collection. With the exception of Svanemyr’s [23] study, all the other studies did not have user involvement in the study’s design.

**Table 2:** Quality assessment of included studies using the QATSDD

QATSDD/Study	1	2	3	4	5	6	7	8	9	10	11	12	13	14	15	16	Total score	%	Grade
1. Wado et al, 2019 [21]	2	3	3	3	3	3	0	3	0	3	N/A	3	3	N/A	0	3	32/42	76.2	Good
2. Blystad et al, 2020 [20]	0	3	2	2	3	2	0	2	N/A	N/A	3	3	0	0	0	3	23/42	54.7	Good
3. Svanemyr, 2019 [23]	0	3	2	2	3	2	0	2	N/A	N/A	3	3	2	1	3	3	29/42	69.0	Good
4. Munakampe et al, 2021 [22]	0	3	3	3	3	2	0	2	0	3	N/A	3	3	N/A	0	3	28/42	66.7	Good
5. Menon et al, 2018 [24]	0	3	3	3	3	2	0	3	0	3	2	3	0	0	0	2	27/48	56.3	Good
6. Austrian et al, 2019 [25]	0	3	3	2	3	2	0	1	0	2	3	3	3	3	0	3	31/48	64.6	Good

0 = criterion not mentioned at all; 1 = criterion very slightly mentioned; 2 = criterion moderately mentioned; 3 = criterion fully explained

*N/A* criterion not applicable

### Prevalence of adolescent pregnancy in Zambia

The prevalence of adolescent pregnancy ranged from 29% in the study using the 2014 ZDHS [21] to 48% in a study in Petauke, Chadiza, and Katete districts in Eastern province [24]. Additionally, prevalence of adolescent motherhood ranged from 20.81% in the Zambezi district, North-Western province [26] to 23.1% in the study using 2014 ZDHS data [22].

The prevalence of adolescent pregnancy in Zambia continues on an upward trajectory according to Zambia’s recent Demographic and Health Surveys. For instance, the total adolescent pregnancy rate declined from 33.8% in 1992 to 30.7% in 1996, then increased to 31.6% in 2002. Table 3 shows an increase from 28.5% in 2014 to 29.2% in 2018 [10]. The Southern (42.5%), Western (41.2%), Eastern (39.5%), North Western (35.7%), and Central (30.6%) provinces recorded higher prevalence than the national average [10].

**Table 3:** Teenagers pregnancy or childbearing in Zambia 1992–2018

Background characteristic	Year					
	**1992**	**1996**	**2002**	**2007**	**2014**	**2018**
**Age**						
15	5.3	4.5	4.5	5.8	4.9	6.4
16	14.7	15.3	15.0	16.2	11.9	15.1
17	29.9	28.3	33.8	28.7	25.7	30.0
18	54.3	46.1	44.2	41.0	41.7	41.9
19	65.6	59.4	56.9	54.6	58.9	52.9
**Residence**						
Urban	28.5	26.6	27.1	20.4	20.0	19.3
Rural	40.0	34.4	34.9	35.0	36.4	37.0
**Province**						
Central	39.8	32.3	32.3	29.3	29.9	30.6
Copperbelt	28.0	29.9	26.4	20.1	16.3	21.0
Eastern	43.7	35.0	35.4	29.7	35.4	39.5
Luapula	36.1	22.6	36.5	32.1	27.9	29.0
Lusaka	30.5	28.6	28.9	20.8	23.8	14.9
Muchinga	-	-	-	-	29.6	29.3
Northern	36.7	31.4	30.6	26.6	30.0	25.9
North Western	31.9	38.0	33.3	37.3	41.0	35.7
Southern	34.4	33.5	31.9	35.9	36.0	42.5
Western	36.4	32.7	39.7	43.6	40.4	41.2
**Education**						
None	45.4	48.1	45.6	54.3	53.2	41.9
Primary	36.5	33.1	35.7	32.9	35.9	36.3
Secondary	21.2	21.1	21.4	20.8	23.1	22.8
**Total**	**33.8**	**30.7**	**31.6**	**27.9**	**28.5**	**29.2**

Source: ZDHS 1992–2018

Prevalence was also observed to be positively correlated with increasing age, occasioning a distinct possibility of increasing vulnerability to risky sexual behaviours on the part of adolescents [1]. We observed a high prevalence among those in rural areas compared to those in urban areas which is indicative of rural–urban disparities from 1992 to 2018. Adolescents in urban areas may have access to improve economic opportunities and social services including SRH services and information compared to those in rural settings.

Also, prevalence of adolescent pregnancy was observed to have decreased with increasing levels of education with the trend showing increasing pregnancy for adolescents with primary and secondary education from 1992 to 2018 and decreasing for those with no formal education (Table 3). The level of education is a key determinant of adolescent pregnancy, with prevalence decreasing with increasing level of education. It is, however, a matter of concern that between 1992 and 2018, the teenage pregnancy rates among students in secondary school remained fairly constant (21.2% in 1992 and 22.2% in 2018). It might be of interest to understand reasons for this stagnation, as well as the comprehensive sexuality education packages in schools. This means that increasing access to education at all levels will help limit adolescent pregnancy [23].

### Factors associated with adolescent pregnancy in Zambia

Of the six papers selected, varying associated factors were identified. These factors are consistent with the individual, socio-economic, environmental, and policy levels of the SEM (Table 4).

**Table 4:** Factors influencing adolescent pregnancy in Zambia

Authors, Year	Aim	Study design	Study Site	Sample size	Population	Factors
**Individual Factors**						
Wado et al. 2019 [21]	Identify contextual factors that influencing adolescent pregnancy and early motherhood in five East African countries	Cross-sectional Secondary analysis	East Africa (Kenya, Tanzania, Uganda, Malawi, Zambia)	21,925 (3675 Zambia)	Adolescent girls (15–19 years)	Age (16 years(OR = 1.63, 95%CI = 1.05–2.53), 17 years(OR = 3.53, 95%CI = 2.28–5.47), 18(OR = 6.55, 95%CI = 4.27–10.05), 19 years(OR = 16.56, 95%CI = 10.52–26.07)), Age at first sex (No sex (OR = 0.22, 95%CI = 0.14–0.34), 5–14 (OR = 4.01, 95%CI = 2.51–6.63), 15–17 (OR = 3.21, 95%CI = 2.13–4.96)), Exposure to media (two of 3 sources(OR = 0.58, 95%CI = 0.43–0.80), all three sources (OR = 0.44, 95%CI = 0.28–0.67)),
Svanemyr 2019 [23]	Explore how gendered sexual norms make young unmarried girls vulnerable to unintended pregnancies in a specific context	Qualitative	Southern province	73	Youth (13–20 years)	Lack of resources, Insufficient knowledge about sexuality and reproduction
Munakampe et al. 2021 [22]	Determine factors affecting the fertility of adolescents aged 15–19 years in Zambia and possible drivers of adolescent fertility	Cross-sectional Secondary analysis	Zambia DHS	3666	Adolescent girls (15–19 years)	Age (16 years (OR = 2.3, 95%CI = 1.3–4.2), 17 years (OR = 6.4, 95%CI = 3.6–11.5), 18 years (OR = 15., 95%CI = 8.9–27.1), 19 years (OR = 29.1, 95%CI = 16.9–50.1), Knowledge of contraception (Knows (OR = 5.4, 95%CI = 1.9–15.6), Contraceptive use (use at least one method (OR = 14.4, 95%CI = 9.2–22.4))
Menon et al. 2018 [24]	Explore factors in the social and cultural environment shaping young people’s sexual behaviour, with specific attention to teenage pregnancy and child marriage in Eastern Zambia	Exploratory mixed method	Eastern Province (Petauke, Chadiza, Katete districts)	1434	Youth (15–24 years)	Early/child marriage, early sexual debut, Limited knowledge and use of contraceptives
Austrian et al. 2019 [25]	Explore transactional sex as a driver of adolescent pregnancy	Mixed method	Lusaka, Central, Copperbelt, and North-Western Provinces	5331	Adolescent girls (10–19 years)	Transactional sex, Multiple sexual partners, Unprotected sex
**Socio-Economic**						
Svanemyr 2019 [23]	Explore how gendered sexual norms make young unmarried girls vulnerable to unintended pregnancies in a specific context	Qualitative	Southern province	73	Youth (13–20 years)	Poverty, Low education level, Peer pressure
Wado et al. 2019 [21]	Identify contextual factors that influencing adolescent pregnancy and early motherhood in five East African countries	Cross-sectional Secondary analysis	East Africa (Kenya, Tanzania, Uganda, Malawi, Zambia)	21,925 (3675 Zambia)	Adolescent girls (15–19 years)	Relationship to household head (spouse OR = 14.79, 95%CI = 11.55–8.63)), Education (Secondary and above (OR = ,0.35 95%CI = 0.16–0.76)), Household wealth (Richest OR = 0.47, 95%CI = 0.26–0.83),
Munakampe et al. 2021 [22]	Determine factors affecting the fertility of adolescents aged 15–19 years in Zambia and possible drivers of adolescent fertility	Cross-sectional Secondary analysis	Zambia DHS	3666	Adolescent girls (15–19 years)	Education (Junior secondary (OR = 0.4, 95%CI = 0.2–0.7), Senior secondary (OR = 0.1, 95%CI = 0.0–0.2), Tertiary (OR = 0.1, 95%CI = 0.0–0.8)), Marital status (married (OR = 6.7, 95%CI = 4.9–9.2)), Household wealth (Poor (OR = 1.7, 95%CI = 1.3–2.4))
Menon et al. 2018 [24]	Explore factors in the social and cultural environment shaping young people’s sexual behaviour, with specific attention to teenage pregnancy and child marriage in Eastern Zambia	Exploratory mixed method	Eastern Province (Petauke, Chadiza, Katete districts)	1434	Youth (15–24 years)	Poverty
Blystad et al. 2020 [20]	Expand understanding on socio-cultural and structural dynamics associated with early pregnancy and school dropout	Qualitative	Southern and Central Provinces (Mazabuka, Chikankata, Monze Chibombo and Kapiri Mposhi)	61	Adolescents, parents, teachers, community leaders, and health workers	School dropout, Poverty
**Environmental Factors**						
Svanemyr 2019 [23]	Explore how gendered sexual norms make young unmarried girls vulnerable to unintended pregnancies in a specific context	Qualitative	Southern province	73	Youth (13–20 years)	Norms governing contraceptive use
Menon et al. 2018 [24]	Explore factors in the social and cultural environment shaping young people’s sexual behaviour, with specific attention to teenage pregnancy and child marriage in Eastern Zambia	Exploratory mixed method	Eastern Province (Petauke, Chadiza, Katete districts)	1434	Youth (15–24 years)	Socio-cultural, gender and sexual norms
**Policy Factors**						
Menon et al. 2018 [24]	Explore factors in the social and cultural environment shaping young people’s sexual behaviour, with specific attention to teenage pregnancy and child marriage in Eastern Zambia	Exploratory mixed method	Eastern Province (Petauke, Chadiza, Katete districts)	1434	Youth (15–24 years)	Lack of access to SRH information and services

Bronfenbrenner’s [19] socio-ecological model/framework posits a multiplicity of factors associated with adolescent pregnancy operationalize at four levels: the individual (microsystem); interpersonal (mesosystem); environmental (ecosystem) and policy level (macrosystem).This model offers explanations regarding the influence of social environments on human development and provision of health services to guide the research effort focusing on key actors/variables which include: age, sex, gender issues, employment status, residence, religion, early marriage, poverty, education level, peer pressure, coercive sexual relations, comprehensive sexuality education, access to sexual and reproductive health services including use contraceptives.

At the Microsystem (individual) level of influence, the adolescent is the main centre of focus in terms of factors affecting responses advocating refraining from indulging in risky sexual behaviours, including having multiple sexual partners. Menon et al. and Wood et al. [24, 27] attest, through various studies carried out, that risky sexual behaviour can be construed as a function of age at sexual debut [21, 24], exposure to media [21], knowledge of sexuality and reproduction [23], knowledge of contraception [22, 24], contraceptive use [22, 24], risky sexual behaviours (transactional sex [25], multiple sexual partners [25], child marriage [24], low levels of education, attitude/ perceptions by the adolescent as well as poverty in the household to which the individual belongs.

At the Mesosystem (interpersonal/socio-economic) level of influence, the individual’s disposition towards sexual behaviour is mostly influenced by close family members without ruling out other factors/variables such as educational attainment [20–23], marital status [22], household wealth/poverty [20, 22–24, 26], peer pressure [23], and relationship to household head [21]. Low household wealth was a major factor identified in many studies [21, 22, 24]. The studies reported that low household economic status increases the risk of adolescent pregnancy with adolescents from the wealthiest households being protected from adolescent pregnancy. Menon et al. note that nationally, household poverty increases the risk of pregnancy among adolescents in Zambia using the 2014 ZDHS data [24].

It was also reported that major factors to adolescent pregnancy at the socio-economic factors were peer pressure [23] and relationship to household head (spouse) [21]. The researchers noted that adolescents who were spouses to the household head were more likely to get pregnant (OR = 14.79, 95%CI = 11.55–8.63) [21]. This however begs the question pertaining to the period the adolescent became pregnant as it is not abundantly clear whether conception occurred before or after marriage to the household head (presumably male-headed household).

Ecosystem (Community/Societal) level factors for adolescent pregnancy were also reported. Key at this level of influence, are neighbours, family and friends who potentially can influence an adolescent to be favourably disposed towards a certain trajectory. It is also at this level, that differences in prevalence rates become glaring as reported by Menon et al. [24]. These were socio-cultural, gender and sexual norms [21, 22] which encouraged early or child marriage, early sexual initiation, unprotected sex, and barriers to accessing sexual and reproductive health information and services. For instance, Svanemyr observes that socio-cultural norms about the use of contraceptive increases the risk of adolescent pregnancy in Zambia [23].

The Macrosystem (Policy/Legal) level of influence can be considered to be the most influential factor as enshrined in legal statutes which have force and effect of law, as attested for instance, by regulatory and policy framework on early marriages. Policy level factors comprised limited accessibility to SRH information and SRH services, including laws which prohibit adolescents from accessing SRH services including contraceptives [24]. The authors observed that due to limited access to sexual and reproductive health information and services many adolescents in Zambia are at risk of unplanned pregnancies.

## Discussion

Given the above contextual background the review then assessed the prevalence and factors influencing adolescent pregnancy in Zambia. It showed that prevalence of adolescent pregnancy varied between provinces as well as nationally. The factors may be categorized as being individual, socio-economic, environmental, and policy level factors.

### Prevalence of adolescent pregnancy

The review revealed a significantly high prevalence of adolescent pregnancy ranging from 29% nationally [21] to 48% in the Eastern and Southern provinces [24]. Some of the studies [21, 22] also assessed adolescent motherhood, which presents as a limitation to capturing the magnitude of the problem, since this approach does not cover adolescents who may have lost pregnancy due to induced abortion or miscarriage. Nevertheless, this prevalence is in tandem with the high prevalence of the national adolescent pregnancy reported in the 2018 ZDHS [10]. It is important to understand the context-specific drivers of adolescent pregnancy in the country. One way could be through carrying out robust country-wide studies to allow for comparisons. This would address information gaps relating to possible ineffective or weak implementation of existing policies and interventions instituted to address the problem [28–30].

### Individual level factors

This review showed that low level exposure to media is a factor significantly impacting adolescent pregnancy in Zambia. Exposure to various media platforms provides SRH information to young people [31, 32], and in some cases, a useful source of information on where to obtain SRH services including contraceptives and abortion services, hence contributing to the removal of some existing barriers to accessing SRH care. Further, exposure to the media has been found to trigger discussions about ASRH issues in addition to encouraging adolescents to utilize SRH youth reproductive and family planning services [33]. Exposure to mass media, can greatly influence adolescents’ attitudes, social expectations and avoidance of risky sexual behaviours thereby reducing risk of pregnancy [31].

Insufficient knowledge about sexual and reproductive health has been noted to increase the risk of adolescent pregnancy in Zambia. This is in line with the findings of many studies carried out in low- and middle-income countries (LMICs) [34–38]. Lack of knowledge about SRH issues indicates prevalence of poor safe sexual practices including contraceptive use and STIs prevention. As such, age-appropriate sexual health education programmes for adolescents are key to developing safe SRH as well as preventing adolescent pregnancy [39].

Early or child marriage was noted to be another a factor impacting Zambia’s adolescent pregnancy as attested by similar findings in previous studies across Africa pointing out the influential role of socio-cultural norms and practices in most developing countries [3, 4, 39]. Child marriage is still prevalent across Africa despite most countries having laws prohibiting and criminalizing marriage before the age of 18 years which might be due to sporadic or total lack of enforcement of these laws with no sanctions when they are routinely violated [40].

It is common cause that risky sexual behaviours among most adolescents have been reported as factors that increase the risk of adolescent pregnancy [27, 41]. Frequent sexual activity at an early age, multiple sexual partners, transactional and unprotected sex are common among adolescents and young people. Such risky sexual behaviours can be attributed, amongst other factors, to household poverty levels as noted in many studies [27, 41, 42]. In fact, a number of studies have reported a tendency among some adolescent girls to use sexual relationships with older men to cater for some of their financial needs [41, 42]. Early sexual initiation and lack of contraceptives as factors impacting adolescent pregnancy, as reported elsewhere in this review, is corroborated by findings from previous studies [3, 43]. In contrast, Worku et al.’s study indicated that contraceptive use among adolescents tends to increase the risk of adolescent pregnancy [44]. This could be explained as result of failed contraceptive failure due to poor knowledge on its correct use.

### Interpersonal/socio-economic level factors

This review also accentuates the importance of two main interpersonal level factors, namely, peer pressure and relationship to household head. At the interpersonal level, supportive relationships that reinforce positive sexual and reproductive health behaviours among adolescents such as family influence, have an impact on the sexual and reproductive health vulnerabilities and experiences of adolescents [45]. Peer pressure as a major factor contributing to adolescent pregnancy has been reported in many other studies in Africa [46–48]. It has been established that peer groups play a significant role as agents of socialization which often influences lifestyle choices including sexual behaviours of teenagers. Pressure from this group may be so overwhelming to an extent that teenagers tend to conform to the sexual behaviour norms considered acceptable to their peer group [49].

Regarding the finding that relationship with household head is associated with adolescent pregnancy, this is not inconsistent with the findings by Worku et al. where relation to household head was significantly associated with teenage pregnancy in East Africa [44]. This could be attributed to the fact that some of the adolescents are married to the household head which increases their likelihood of becoming pregnant as wedded adolescents, a fact also identified by Wado et al. [21]. It is worth noting that teenage pregnancy mostly precedes the marriage. This is specially the case in most developing countries where sometimes entering into marriage is occasioned by unplanned pregnancy [50, 51].

Additionally, low level educational attainments were associated with increased risk of adolescent pregnancy in Zambia. This finding resonates well with those of other similar studies which report that low educational expectations and school dropouts are common factors for adolescent pregnancy [8, 43, 52–54]. Studies have found a link between the number of years spent in school and modern contraceptive use with more years associated with higher chances of using modern contraceptives [55–59]. In addition, educational attainment and adolescent pregnancy are interconnected such that while low educational attainment increases risk of adolescent pregnancy, unplanned pregnancy often truncates education for most adolescent girls. This could be attributed to the fact that education empowers adolescent girls regarding their sexual relationships and safe sex practices [60].

Low socio-economic status in the form of poor household wealth, poverty, and lack of resources tend to have a direct and high impact on the risk of adolescent pregnancy. Several studies have indicated that low family incomes increase the risk of adolescent pregnancy [38, 42]. These studies further observed that adolescent girls engaged in sexual relations with men in exchange for gifts and money or in return for financial support for their needs including but not limited to basic necessities such as sanitary pads and school fees [61, 62]. It can be noted that household poverty potentially increases the vulnerability of adolescent girls due to the need to meet some basic necessities. This may be construed as explaining the use of transactional sex as an economic survival strategy by young girls from households with high poverty levels risking pregnancy at a younger age [63].

### Environmental level factors

The finding that existing socio-cultural, gender and sexual norms in Zambia are linked to increased risk of adolescent pregnancy is supported by many other studies [35, 37, 64, 65]. For instance, on cultural beliefs, encouraging larger families and early childbearing, Ayele et al. and Kaphagawani and Kalipeni reported that the prevailing cultural norms of marrying off young girls once they reach puberty leads to adolescent pregnancy [3, 4]. Studies have also found that being a spouse was a significant predictor of adolescent pregnancy [4, 44, 54]. These norms may contribute to adolescent girls seeing unplanned pregnancies as normal and socially accepted.

Similarly, norms governing contraceptive use were found to be a factor in adolescent pregnancy. Community-wide negative perceptions and beliefs regarding modern contraceptives was noted to be a common predictor of adolescent pregnancy in previous studies [66–69]. These norms lead to the unacceptability and non-use of modern contraceptives among adolescents. In some cases, parents and guardians do not support the provision of contraceptive services to adolescents hence recommendations have been made to engage and persuade key community gatekeepers such as religious leaders and chiefs to generate wider community support [66, 70]. In this regard, Svanemyr et al. contend that as part of efforts to reduce environmental level factors, positive social norms and community support need to be enabled for adolescents to practice safer sexual behaviours and have increased access to SRH information and services [45].

### Policy/legal level factors

In addition to the above-mentioned factors, limited access to SRH information and services was identified as a policy/legal level factor for adolescent pregnancy in Zambia. Other studies in LMICs have also reported that lack of SRH service increases the risk of adolescent pregnancy [71–73]. Such a situation hinders adolescents from receiving much-needed SRH information, counselling and contraceptive services [71], against a background where the unmet need for contraceptives alone is considerably high in LMICs [74]. At the policy level, embedded provisions in some laws and policies related to the health, socio-economic, educational sectors and broad societal norms may hinder access to SRH and adolescents’ enjoyment of their human rights and freedoms [45]. In Zambia, adolescents below 16 years of age require parental/guardian consent to access SRH services, especially contraceptives.

The factors identified in this review suggest that the factors associated with adolescent pregnancy are varied but multifaceted and interconnected. There was more evidence about determinants of adolescent pregnancy in the individual and socio-economic levels compared to the environmental and policy levels. This indicates paucity of empirical evidence at these levels to support policy and intervention development and likely cause of the failure of some efforts to reduce the problem of teenage pregnancy. It thus shows that in designing interventions, little evidence from environmental and policy levels have been considered and incorporated. We did not observe any differences in the factors/levels identified in the literature based on the indicators of adolescent pregnancy measured by researchers (record of live birth or self-report). However, studies using primary data captured factors at more levels than those using secondary data.

### Strengths and limitations

This systematic review presents findings of critical importance to the prevention of adolescent pregnancy in Zambia. However, the pulled studies do not cover all the regions, hence limiting the representativeness of the review findings to generalize on the whole country. In some of the included studies, adolescent motherhood was instead assessed as proxy to adolescent pregnancy. This presents as a limitation in capturing the magnitude of the problem since this approach does not cover adolescents who may have lost pregnancy due to induced abortion or miscarriage. In addition to this, the low number of studies included in the final review and the inclusion of various designs of studies that led to the heterogeneity which hindered the ability to perform a meta-analysis. In addition, there was no control over the data collection, cleaning and analysis of the studies considered in this study. There is however, merit on the integrity of the data and findings of the selected studies which are very germane and relevant to the prevention of adolescent pregnancy in Zambia and other SSA countries.

### Implications

The prevalence of adolescent pregnancy in Zambia remains high and could be a major hindrance for the country to attain SDGs on health and wellbeing (SDG3) gender equality and women’s empowerment (SDG5) and decent work (SDG8) [7]. Numerous health interventions such as health education, skill-building, and increasing accessibility to contraceptives [75–78] including policies and legal frameworks have been implemented over the years to improve the health of young people and decrease adolescent pregnancy in the country [79]. Adolescent pregnancy however remains high. These interventions and policies are somewhat fragmented and not designed with multi-level and multi-sectoral connections. Thus, they may tend to overlook other aspects of the multifaceted factors being delivered or may serve to create other avenues which may mitigate against these efforts. Robust and nationally representative studies are needed to fill this lacuna.

## Conclusion

Zambia’s high adolescent pregnancy rates were found to be associated with various individual, socio-economic, environmental, and policy level factors. This suggests that stakeholders need to consider a multilevel and multisectoral approach in addressing the challenge. There a is need to address the socio-cultural dimensions and foster political will in interventions, such as using incentives to stay in school, strategies to delay pregnancy and marriage, which include access to SRH information and services, use of positive deviant approaches in communities, policy/legal review, advocacy at policy level to end child marriages, investment in contraception, and access to adolescent friendly health care services.

## Data Availability

Data sharing is not applicable to this article as no datasets were generated or analysed during the current study. Nevertheless, the selected articles have all been listed in Table 2.
